# Comparative analysis of AAV serotypes for transduction of olfactory sensory neurons

**DOI:** 10.3389/fnins.2025.1531122

**Published:** 2025-02-13

**Authors:** Benjamin D. W. Belfort, Johnathan D. Jia, Alexandra R. Garza, Anthony M. Insalaco, J. P. McGinnis, Brandon T. Pekarek, Joshua Ortiz-Guzman, Burak Tepe, Hu Chen, aSCENT-PD Investigators, Benjamin Arenkiel, Zhandong Liu, Benjamin R. Arenkiel

**Affiliations:** ^1^Genetics and Genomics Graduate Program, Baylor College of Medicine, Houston, TX, United States; ^2^Department of Molecular and Human Genetics, Baylor College of Medicine, Houston, TX, United States; ^3^Jan and Dan Duncan Neurological Research Institute, Texas Children’s Hospital, Houston, TX, United States; ^4^Medical Scientist Training Program, Baylor College of Medicine, Houston, TX, United States; ^5^Aligning Science Across Parkinson’s (ASAP) Collaborative Research Network, Chevy Chase, MD, United States; ^6^Section of Neurology, Department of Pediatrics, Baylor College of Medicine, Houston, TX, United States; ^7^Department of Neurosurgery, Baylor College of Medicine, Houston, TX, United States; ^8^Department of Neuroscience, Baylor College of Medicine, Houston, TX, United States

**Keywords:** AAV, snRNAseq, gene therapy, olfaction, olfactory sensory neurons

## Abstract

Olfactory sensory neurons within the nasal epithelium detect volatile odorants and relay odor information to the central nervous system. Unlike other sensory inputs, olfactory sensory neurons interface with the external environment and project their axons directly into the central nervous system. The use of adeno-associated viruses to target these neurons has garnered interest for applications in gene therapy, probing olfactory sensory neuron biology, and modeling disease. To date, there is no consensus on the optimal AAV serotype for efficient and selective transduction of olfactory sensory neurons *in vivo*. Here we utilized serial confocal imaging and single-nucleus RNA sequencing to evaluate the efficacy of 11 different AAV serotypes in transducing murine olfactory sensory neurons via non-invasive nasal inoculation. Our results reveal that AAV1, while highly effective, exhibited broad tropism, whereas AAV-DJ/8 showed the greatest specificity for olfactory sensory neurons.

## Introduction

The mammalian olfactory epithelium (OE) is a specialized epithelial tissue within the nose that plays a critical role in the sense of smell. Olfactory sensory neurons (OSNs) are specialized chemosensory neurons that reside in the OE and detect airborne chemicals through G-protein coupled odorant receptors. OSNs project their axons directly into the olfactory bulb (OB), creating a single-neuron chain between the external environment and the central nervous system (CNS). This conduit into the CNS provides unique opportunities for probing OSN biology, administering targeted gene therapies, and modeling disease. The use of AAVs has been explored for these purposes (Gadenstaetter et al., 2022), yet information is lacking on the optimal serotype for transducing OSNs. In the present study, we leveraged confocal imaging and single-nucleus RNA sequencing (snRNAseq) to identify optimal AAV serotypes for *in vivo* transduction of murine OSNs.

## Results

**Figure 1:**
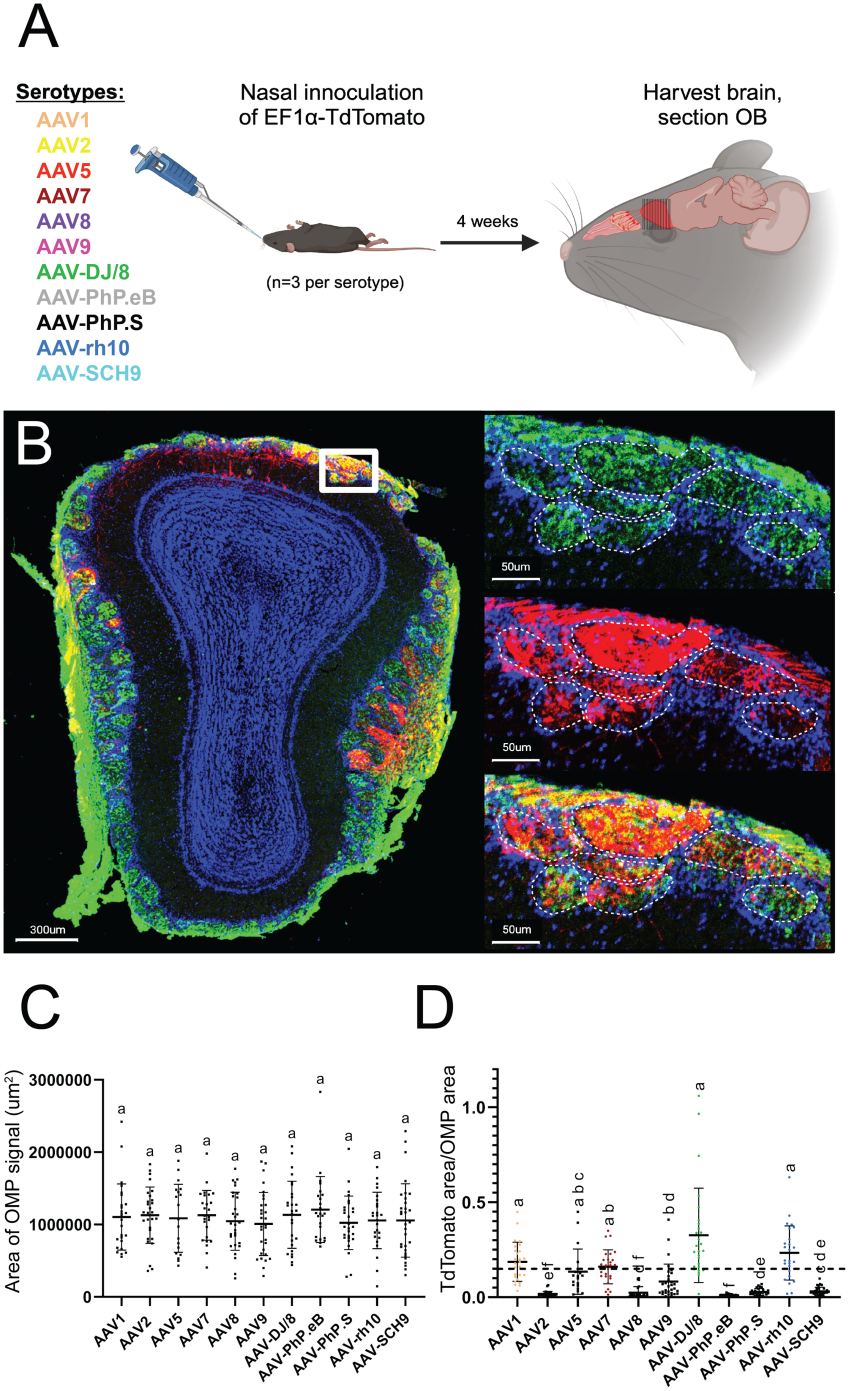
Confocal imaging identifies AAV1, AAV7, AAV-DJ/8, and AAV-rh10 as efficient AAV serotypes for OSN transduction. **(A)** Overview of experimental paradigm. Identical plasmid constructs were packaged in 11 unique AAV serotypes, and each serotype was individually introduced via NINI. **(B)** Representative confocal image of OB cross-section from an AAV1 inoculated mouse. Upper right: close-up of OSNs forming the structures of the glomerular (outlined with dashed white lines) and olfactory nerve layers of the OB, with OMP (green) channel isolated. Middle right: close-up of the same region, with TdTomato (red) channel isolated. Bottom right: overlay of OMP and TdTomato channels. Blue = Hoechst. **(C)** Total area of OMP signal per OB section for each serotype. **(D)** Quantification of the percentage of TdTomato/OMP in the glomerular layer per OB section. Error bars represent the mean with standard deviation. Serotypes with a mean above 15% (horizontal dotted line) are represented by individual coloration, while those below all remain black. Statistical differences were assessed using one-way ANOVA followed by Tukey’s *post hoc* test, and the results are denoted by letters above each bar. Bars sharing a letter are not significantly different from one another (*p* > 0.05).

To test the efficacy of AAV transduction across commonly used serotypes, we packaged an identical construct (rAAV-EF1a-TdTomato-WPRE-PolyA) into 11 different AAV capsid serotypes with reported OE and neuronal transduction (Gadenstaetter et al., 2022). The naturally occurring serotypes used in this study were AAV1, AAV2, AAV5, AAV7, AAV8, and AAV9, while the engineered capsids include AAV-DJ/8 (Grimm et al., 2008; Hammond et al., 2017), AAV-PhP.eB (Chan et al., 2017), AAV-PhP.S (Chan et al., 2017), AAV-rh10 (Cearley and Wolfe, 2006; Gao et al., 2002), and AAV-SCH9 (Ojala et al., 2018). Each AAV serotype was introduced via non-invasive nasal inoculation (NINI) (Santry et al., 2017) into three male mice per serotype, for a total of 33 experimental animals. After a four-week expression period, the olfactory bulb (OB) was processed to assess the co-localization of olfactory marker protein (OMP) immunofluorescence and AAV construct-driven TdTomato expression within OSN axon terminals in the glomerular layer. This approach measures the area of co-localized signal rather than directly counting neurons and serves as a proxy for the number of transduced OSNs (Figure 1A). By focusing on the reporter expression in the glomerular layer of the OB rather than cell body expression in the OE, we avoided potentially confounding TdTomato signals from other transduced accessory cell types within the OE.

For quantification, we performed confocal image analysis across OSN terminal fields within the glomerular layer (Figure 1B). Importantly, we found no difference in total OMP area between samples from each serotype (Figure 1C), suggesting that transduction itself did not compromise overall OSN viability or OE integrity. We next quantified the amount of TdTomato surface area (normalized to OMP surface area) per section, and found that AAV1, AAV7, AAV-DJ/8, and AAV-rh10 displayed the greatest levels of expression (Figure 1D) in OSN terminals that project to the olfactory bulb. Off target transduction within the main olfactory bulb was quantified but found to be insignificant within all serotypes, with the highest count being only 10 cells in a given cross section (Supplementary Figure S1).

**Figure 2:**
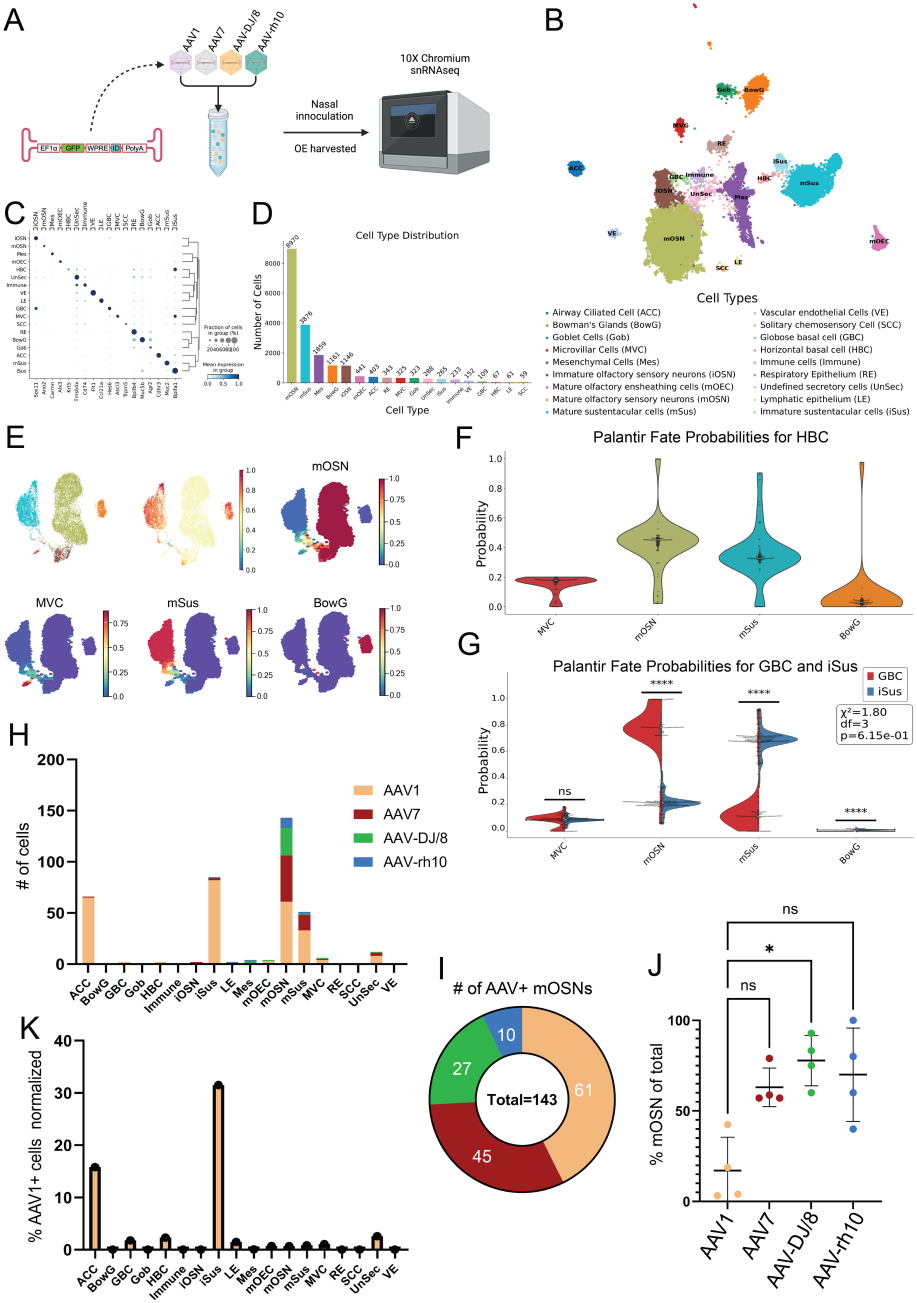
Single nucleus transcriptomics shows AAV1 has the greatest transduction rate while AAV-DJ/8 has the greatest specificity for mOSNs. **(A)** Experimental workflow. **(B)** UMAP of snRNAseq dataset. **(C)** Dot plot showing the cell-type specific markers used for annotations. The *x*-axis is the marker, and the *y*-axis is the cell type. **(D)** Cell type distribution with total cell counts. The *x*-axis is the cell type with each bar’s color reflecting the corresponding color on the UMAP, and the *y*-axis is the absolute count of the respective cell types. **(E)** Minimum distortion embedding (MDE) with our specific lineages of interest including mOSN, mSus, MVC, and BowG lineages. The total OE cell type-specific pseudotime is shown above and individual lineage pseudotimes shown below, with red indicating late pseudotime and dark blue indicating early pseudotime. **(F)** Violin-box plot of terminal fate probability of HBCs calculated from the Palantir pseudotime. **(G)** Terminal fate probabilities of GBCs and iSus were compared using a two-sided *t*-test (*α* = 0.05) and chi-squared goodness of fit for each possible terminal fate. The stars represent the level of significance. If *p* < 1 × 10^−4^ there are 4 stars, <1 × 10^−4^ are 3 stars, 2 stars for <0.01, 1 star for <0.05, and ns for not significant. Chi-squared GOF was used to evaluate overall differences between terminal fate probabilities of each cluster. **(H)** Total AAV+ cell counts per cell type. **(I)** Total AAV+ mOSN cell counts. **(J)** Percent of total serotype positive cells which are mOSNs. ^*^p < 0.05, Kruskal–Wallis test. **(K)** Percent AAV1+ cell counts of each cell type cluster total cell count.

Following this primary reporter expression screen, we next performed snRNAseq to more accurately quantify OSN transduction efficiency of the top four candidates in the different OE cell types. Towards this, we designed four new AAV expression constructs, all identical aside from a short barcode sequence (“ID”) that identifies the corresponding AAV serotype upon transduction and snRNA sequencing. The four AAVs were mixed at equal titer and introduced into wildtype mice via NINI. After 4 weeks of *in vivo* expression, OE was harvested for snRNAseq (Figure 2A). Data was processed following standard scRNAseq guidelines (Wolf et al., 2018). Using 20,081 high-quality nuclei, we generated an annotated UMAP from known cell type-specific markers (Table 1) to quantify the distribution of cell types (Figures 2B–D) and identify those that were differentially transduced by the four candidate AAV serotypes. We confirmed cell type lineages by comparing terminal fate probabilities from Palantir pseudotime analysis, with horizontal basal cells (HBCs) designated as the starting point and mature OSN (mOSN), mature sustentacular (mSus), microvillar (MVC), and Bowman’s gland (BowG) cells as terminal fates (Setty et al., 2019) (Figures 2E,F). A two-sided *t*-test (*α* = 0.05) and chi-squared goodness of fit indicated that the terminal fate probabilities between GBCs and iSus cells were different for mOSN and mSus lineages (Figure 2G). Finally, we quantified the number and type of cells that were effectively transduced for each AAV serotype (Figure 2H). In total, we identified 382 cells that harbored AAV barcodes. While mOSN transduction efficiency was highest for AAV1 (Figure 2I), AAV-DJ/8 showed the greatest specificity for mOSNs (Figure 2J). Further examination of AAV1 expression showed broad tropism, with immature sustentacular (iSus) and airway ciliated cells (ACCs) contributing to the highest normalized cell counts (Figure 2K).

**Table 1:** Cell types and cell type markers.

Cell type	Markers
Airway ciliated cell (ACC)	Foxj1, Cdhr3, Cdhr4
Bowman’s gland (BowG)	Muc5b, Bpifa1, Aqp5, Sox9, Bpifb9b, Bpifb9a
Globose basal cell (GBC)	Sox11, Kit, Neurod1, Neurog1, Hes6, Ascl1
Goblet cell (Gob)	Agr2, Ltf, Spdef
Horizontal basal cell (HBC)	Krt5, Krt14, Krt17, Trp63
Immune cells (Immune)	Cd19, Cd74
Lymphatic epithelium (LE)	Ccl21a
Microvillar cell (MVC)	Ascl3, Cftr, Hepacam2, Lcn11
Mesenchymal cell (Mes)	Alx1, Tfap2b, Carmn, Col1a1, Col1a2, Pdgfra, Vim, Runx2, Pdgfrb
Mature olfactory ensheathing cell (mOEC)	Alx3, Ptprz1, Ptn, Plp1, Mmd2
Immature olfactory sensory neuron (iOSN)	Gap43, Neurod1, Sox11, Dcx, Lhx2, Gng8, Tubb3, Stmn1, Stmn2
Mature olfactory sensory neuron (mOSN)	Ano2, Olfm1, Chga, Kcnk10, Cnga2, S100a5, Gnai
Respiratory epithelium (RE)	Bpifb4
Solitary chemosensory neuron (SCC)	Trpm5
Immature sustentacular cell (iSus)	Bpifa1, Mybl1, Lgr5
Mature sustentacular cell (mSus)	Muc2, Cyp2g1, SLc2a3, Ackr3
Undefined secretory cell (UnSec)	Tmsb4x, Scgb1c1, S100a5,
Vascular endothelial cell (VE)	Vwf, Flt1

## Discussion

In the present study, we leveraged confocal imaging and snRNAseq to identify optimal AAV serotypes for *in vivo* OSN transduction via NINI. Despite variability in our imaging dataset, attributable to unpredictable flow of fluid through the nasal turbinates during NINI, AAV1, AAV7, AAV-DJ/8, and AAV-rh10 displayed the greatest efficacy in their ability to transduce OSNs compared to other AAV serotypes we tested (Figure 1D). To determine which serotype was most efficient at transducing OSNs in a more quantitative, higher-resolution, and cell type-specific manner, we employed snRNA sequencing from isolated OE. We chose snRNAseq over traditional scRNAseq to maximize the sequencing depth per cell, as snRNAseq focuses on nuclear transcripts and reduces the total transcript pool, thereby increasing the likelihood of detecting rare transcripts, such as those derived from AAV genomes. While this approach may limit the total number of transcripts detected compared to scRNAseq, it was intentionally selected to optimize the sensitivity for identifying AAV-derived transcripts within the constraints of our sequencing depth. Ultimately, our study identified relatively few transduced cells suggesting either low overall transduction efficiency or missing counts from dropout due to low sequencing depth, which we are unable to disentangle. Additionally, identifying an ideal promoter optimized for robust and specific transgene expression in OSNs is an important consideration, as promoter efficiency may significantly influence both transduction outcomes and detection sensitivity. Though this is the first study to employ snRNAseq of the OE, we found that clustering and cell type distribution were similar to other single-cell sequencing studies previously performed on the OE (Brann et al., 2020; Fletcher et al., 2017; Li et al., 2024) (Figures 2B–D). We found that cell subsets comprised known lineages of interest, namely HBCs, globose basal cells (GBCs), immature OSNs (iOSNs), mOSNs, iSus, mSus, MVCs, and BowGs, and determined the corresponding pseudotemporal lineage patterns (Figure 2E). Through this, we substantiated cell type identities by comparing their terminal fate probabilities. Of interest, the terminal fate probability distribution of HBCs properly reflected multiple cell types, indicating their pluripotent ability to divide into all lineages of interest, but favoring mOSNs, mSus, and MVCs (Figure 2F). By comparing the terminal fates of iSus and GBC, we further validated the identified OSN and sustentacular cell lineages. Having confirmed the cellular composition of the transduced tissue, we next quantified the number of AAV+ cells within each cell type cluster (Figure 2H). Of the serotypes tested, AAV1 showed the highest transduction efficiency of OSNs (Figure 2I), but AAV-DJ/8, AAV7, and AAV-rh10 all showed greater OSN specificity, with AAV-DJ/8 showing the greatest specificity compared to AAV1 (Figure 2J). From these data, we also found that AAV1 showed the broadest tropism in the OE; transducing cell types such as HBCs, GBCs, ACCs, and particularly iSus, all with greater efficiency than OSNs (Figure 2K). Alongside a census of the cell types that comprise the mouse olfactory epithelium, together these findings also present useful implications for future research, including OSN-targeted gene therapy, probing OSN biology, modeling diseases where the OE and OB are loci of interest (e.g., Parkinson’s disease and Alzheimer’s disease), and informing the development of future AAV capsids for targeting OSNs.

## Materials and methods

### Animals

All mice used in this study were male C57BL/6J (Jax: 000664) and were used in compliance with Baylor College of Medicine IACUC. For single serotype inoculations, mice were 8 to 9 weeks old, and for combined serotype inoculations, mice were 10 weeks old.

### Plasmid constructs and nasal inoculation

For all nasal inoculations, mice were briefly anesthetized with isoflurane. While under anesthesia, the mice were nasally inoculated in 5 μL doses for a total of 40 μL (alternating between nostrils, with 20 μL of virus per nostril).

For inoculations of individual serotypes, rAAV-Ef1α-TdTomato-WPRE-polyA was packaged in serotypes AAV1, AAV2, AAV5, AAV7, AAV8, AAV9, and the engineered capsids AAV-DJ/8, AAV-PHP.eB, AAV-PHP.S, AAVrh10, or AAV-SCH9. The plasmid was used as a template during the packaging process, with only the transgene and necessary regulatory elements being encapsulated within the AAV capsid, while the plasmid backbone remains excluded. Each serotype preparation was normalized to a final concentration of 4.85 × 10^11^ vg/mL (aa total of 1.94 × 10^10^ vg per serotype). Three mice were inoculated with each individual serotype, for a total of 33 mice.

For mixed inoculations, unique variants of an rAAV-Ef1α-EGFP-WPRE-ID-PolyA construct were packaged in AAV1, AAV7, AAV-DJ/8, and AAVrh10, with unique barcodes (ID) corresponding to each serotype. The AAVs were combined in equal concentrations (final concentration of 1.22 × 10^11^ vg/mL, a total of 6.10 × 10^9^ vg per serotype). The IDs are listed below:

AAV1 ID: CGACGCCTTGTGGATTTTCGTTTTA
AAV7 ID: CGACGATTTTCGTTTTACCTTGTGG
AAV-DJ/8 ID: CGACGCGTTTTACCTTGTGGATTTT
AAVrh10 ID: CGACGGATTTTCGTTTTACCTTGTG

For materials and methods regarding AAV production, please refer to the Supplementary material. For a complete list of key reagents and resources, refer to Table 2.

**Table 2:** Key resources.

Reagent type (species) or resource	Designation	Source or reference	Identifiers	Additional information
Strain, strain background (*Mus musculus*, *male*)	C57BL/6J	The Jackson Laboratory	RRID:IMSR_JAX:00066	8–9 weeks old for single serotype inoculations, 10 weeks old for combined serotype inoculations
Chemical compound, drug	OptiPrep	Millipore Sigma	D1556-250ML	Also known as iodixanol
Chemical compound, drug	OptiSeal 16 × 67 mm tubes	Beckman Coulter	Cat# 362181	Loaded with the iodixanol gradient
Other	NVT 65 Near-Vertical Rotor	Beckman Coulter	Cat# 362755	Centrifuged at 60,000 rpm for 90 min
Other	Amicon Ultra-15 Centrifugal Filter	Millipore Sigma	UFC910024	For OptiPrep removal and AAV concentration
Commercial assay, kit	qPCR AAV Titer Kit	Applied Biological Materials	Cat# G931	For viral titer
Other	AAV production	This paper	dx.doi.org/10.17504/protocols.io.81wgbzwj3gpk/v1	For production of the 11 serotypes described
Recombinant DNA reagent	rAAV-Ef1α-TdTomato-WPRE-polyA	This paper		Packaged into the 11 AAV serotypes described
Recombinant DNA reagent	rAAV-Ef1α-EGFP-WPRE-ID-PolyA	This paper		AAV1 ID: CGACGCCTTGTGGATTTTCGTTTTA AAV7 ID: CGACGATTTTCGTTTTACCTTGTGG AAV-DJ/8 ID: CGACGCGTTTTACCTTGTGGATTTTA AVrh10 ID: CGACGGATTTTCGTTTTACCTTGTG
Chemical compound	16% PFA diluted in PBS	Electron Microscopy Sciences	Cat# 15710	Diluted to 4% in 30 mL PBS
Chemical compound	Tissue-Plus O.C.T. Compound	Fisher Scientific	Cat# 23-730-571	
Other	CM1860 Cryostat Microtome	Leica Biosystems		40 μm sections
Antibody	Anti-OMP (Goat, polyclonal)	FUJIFILM Wako	Cat# 544-10001-WAKO	Immunofluorescence (1:20,000)
Antibody	Anti-Goat Alexa Fluor 488 (Donkey, polyclonal)	Invitrogen	Cat# A-11055	Immunofluorescence (1:1,000)
Chemical compound, drug	Hoechst	Thermo Fisher Scientific	Cat# 62249	Immunofluorescence (1:1,000)
Chemical compound, drug	Fluoromount-G	SouthernBiotech	Cat# 0100-01	Tissue mounting media
Other	TCS SP8 confocal Microscope	Leica Biosystems		Equipped with 20×/0.75 objective
Software, algorithm	LAS X	Leica Biosystems	RRID:SCR_013673	Imaging software for the confocal microscope
Software, algorithm	Imaris v10.0	Bitplane	RRID:SCR_007370	Analysis of OB sections
Other	Image analysis	This paper	dx.doi.org/10.17504/protocols.io.n2bvjn1rxgk5/v1	For quantifying TdTomato expression in OB axon terminals
Chemical compound, drug	Nuclei Extraction Buffer	Miltenyi Biotec	Cat# 130-128-024	Lysis buffer
Chemical compound, drug	Protector RNAse Inhibitor	Millipore Sigma	Cat# 3335402001	
Other	GentleMACS C tubes	Miltenyi Biotec	Cat# 130-093-237	
Other	GentleMACS Octo Dissociator	Miltenyi Biotec	Cat# 130-096-427	
Other	MACS SmartStrainers 70 μm	Miltenyi Biotec	Cat# 130-098-462	
Other	MACS SmartStrainers 30 μm	Miltenyi Biotec	Cat# 130-098-458	
Commercial assay, kit	Chromium Single Cell 3′ Reagent Kits User Guide (v3.1 Chemistry)	10X Genomics	PN-1000121	Single-cell library preparation
Software, algorithm	STAR v2.7.11b	Thomas Gingeras Lab	RRID:SCR_004463	Align paired-end snRNAseq FASTQ files
Software, algorithm	STARSolo	Alexander Dobin Lab	RRID:SCR_021542	Align paired-end snRNAseq FASTQ files
Ssoftware, algorithm	GRCm39 vM34	GENCODE	RRID:SCR_014966	Custom FASTA and GTF file creation
Software, algorithm	CellBender v0.3.0	Broad Institute	https://doi.org/10.1038/s41592-023-01943-7	Empty droplet filtering
Software, algorithm	Python v3.10.14	The Python Software Foundation	RRID:SCR_008394	
Software, algorithm	Scanpy v1.10.1	Fabian Theis Lab	RRID:SCR_018139, https://github.com/scverse/scanpy	Processing and QC of single-cell data
Software, algorithm	Solo (scvi-tools v1.0.4)	Nir Yosef Lab	https://github.com/scverse/scvi-tools	Doublet removal
Software, algorithm	Rapids single-cell v0.10.5	Nir Yosef Lab	https://rapids-singlecell.readthedocs.io	GPU-acceleration of Scanpy processing steps
Software, algorithm	pyMDE v0.1.18	Minimum-distortion embedding, 2021	https://pymde.org	MDE generation
Software, algorithm	Palantir v1.3.3	Dana Pe’er Lab	https://github.com/dpeerlab/Palantir	Pseduotime determinations
Software, algorithm	RNA analysis code	This paper	https://doi.org/10.5281/zenodo.13620762	Generated in house

### Tissue processing for immunofluorescence

Four weeks after nasal inoculation with individual AAV serotypes, the 33 mice were deeply anesthetized with isoflurane and transcardially perfused with 10 mL of ice-cold PBS followed by 10 mL of 4% paraformaldehyde (16% PFA diluted in PBS; Electron Microscopy Sciences 15710). The olfactory bulbs (OBs) were then harvested, fixed in 4% PFA at 4°C overnight, and moved to 30% sucrose (36 h) for cryoprotection. OBs were frozen in OCT (Fisher HealthCare cat# 23-730-571) and sectioned at 40 μm on a cryostat (Leica CM1860), with every third section collected for analysis. Tissue sections were blocked in 5% Donkey Serum (1X PBS, 0.1% Triton) for 1 h at room temperature, washed three times in PBST (0.1% Triton) and incubated with anti-OMP (1:20,000, FUJIFILM Wako Chemicals U.S.A. Corporation cat# 544-10001-WAKO) antibody at 4°C overnight. Sections were then washed three times with PBST (0.1% Triton), incubated with secondary antibody (anti-goat, Alexa Fluor 488 Invitrogen cat# A-11055) for 1 h at room temperature, washed three times with PBST, incubated with Hoechst (1:1,000, Thermo Fisher cat# 62249) for 15 min at room temperature, and finally washed one more time with PBST before being covered with Fluoromount-G (Southern Biotech cat# 0100-01) and sealed for microscopy.

### Microscopy and image analysis

OB images were taken using a Leica TCS SP8 confocal microscope equipped with a 20×/0.75 objective and Leica LAS X software (RRID:SCR_013673, https://www.leica-microsystems.com/products/microscope-software/details/product/leica-las-x-ls). To obtain the entire volume of each OB section, Z-stacks of ~30–40 μm per sample were collected at 1024 × 1024 resolution with 4× line averaging and 2× frame averaging. All images were stitched within the LAS X software. OB images were analyzed using Imaris software (v10.0, RRID:SCR_007370, http://www.bitplane.com/imaris/imaris). For each image, a region of interest was drawn over the glomerular layer (excluding the olfactory nerve layer) and was used to generate a mask. Volumetric surfaces were then generated following absolute intensity thresholding to account for per-sample background fluorescence signal. To quantify transduction efficiency of each serotype, the total area (μm^2^) of TdTomato signal and OMP signal within the mask were quantified. The ratio of TdTomato signal to OMP signal was then calculated. An average of 23.3 OB sections were imaged for each of the 33 mice, for a total of 760 confocal images.

To blind the researchers to which serotype was being analyzed, image files were automatically renamed and randomly sorted into separate folders using a program generated in house. The files were converted back into their identifiable names after all Imaris-based quantification was completed.

For a more comprehensive guide on how to analyze images in this manner, please refer to the following protocols.io DOI: dx.doi.org/10.17504/protocols.io.n2bvjn1rxgk5/v1.

To access the complete imaging dataset, please refer to “Comparative Analysis of AAV Serotypes for Transduction of Olfactory Sensory Neurons,” accession number S-BIAD1370 (DOI: 10.6019/S-BIAD1370), on BioImage Archive.

### Tissue harvest and nuclei isolation

Four weeks after nasal inoculation with AAV1, AAV7, AAV-DJ/8, and AAVrh10 (mixed together in equal proportions, final concentration of 1.22 × 10^11^ vg/mL for each serotype), four male C57BL/6J mice were deeply anesthetized and transcardially perfused with ice-cold PBS. The OE was then rapidly collected. Immediately following dissection, samples were cut into small pieces and processed using GentleMACS nuclei isolation protocol [Nuclei Extraction Buffer (Miltenyi Biotec, cat# 130-128-024), Protector RNAse Inhibitor (Millipore Sigma, cat# 3335402001), GentleMACS C tubes (Miltenyi Biotec, cat# 130-093-237), GentleMACS Octo Dissociator (Miltenyi Biotec, cat# 130-096-427), MACS SmartStrainers 70 μm (Miltenyi Biotec, cat# 130-098-462), MACS SmartStrainers 30 μm (Miltenyi Biotec, cat# 130-098-458)]. In brief, samples were placed in 2 mL of Miltenyi Nuclei Isolation Buffer and Protector RNAse Inhibitor in GentleMACS C tubes. Samples then underwent the preprogrammed “nuclei isolation” program in a GentleMACS Octo Dissociator. Immediately after dissociation, samples were strained through a 70 μm MACS SmartStrainer and collected in a 15 mL tube and centrifuged at 500 × g for 5 min at 4°C. The supernatant was extracted and discarded, and the resulting pellet resuspended in 1 mL of ice-cold PBS. Resuspended samples were then run through a 30 μm MACS SmartStrainer. Upon visual inspection of nuclei following isolation, it was determined that debris levels were low enough for samples to proceed immediately to library preparation.

For additional information regarding snRNAseq library preparation and sample submission parameters, please refer to the Supplementary material.

### snRNAseq bioinformatic analysis

#### Alignment and quantification of transcripts

Four sets of paired-end snRNAseq FASTQ files, each corresponding with a technical replicate, were aligned and quantified using STAR v2.7.11b (RRID:SCR_004463, http://code.google.com/p/rna-star/) and STARSolo (RRID:SCR_021542, https://github.com/alexdobin/STAR/blob/master/docs/STARsolo.md) (Kaminow et al., 2021). For STARSolo to generate the spliced/unspliced count matrix, we used the velocyto flag available in the pipeline. All downstream steps were performed using the spliced count matrix unless specified otherwise. To include the AAV serotypes in the alignment, we generated a custom reference FASTA and GTF file using the GENCODE (RRID:SCR_014966, https://www.gencodegenes.org) GRCm39 vM34 release (Frankish et al., 2023).

#### Ambient RNA correction

After alignment and quantification, raw spliced, unspliced, and ambiguous count matrices were generated for each sample. To reduce the effects of ambient RNA contamination and filter out empty droplets, we applied CellBender v0.3.0 to each replicate’s corresponding spliced/mature RNA count matrix (Fleming et al., 2023). The resulting corrected matrix was transformed into a Scanpy object for further analysis in Python v3.10.14 (RRID:SCR_008394, https://www.python.org).

#### Processing, quality control, and integration of snRNAseq data

Each replicate was processed and filtered according to the standard processing guidelines from Scanpy v1.10.1 (RRID:SCR_018139, https://github.com/theislab/scanpy) (Wolf et al., 2018). Initial thresholds of minimum 10 cells and 200 genes were set to remove any empty droplets missed by CellBender. Gene/feature initial cutoffs were left more lenient to avoid any possibility of filtering out the AAV genes in case low transduction and expression in the dataset. We set feature/gene and UMI barcode cutoffs to remove dead, low-quality cells, and doublets/multiplets. The cutoffs were set as: 1,500 for number of features, 2,000 for total counts, and 1% for percent mitochondrial counts. Any cells with gene, count, and mitochondrial count percentages above these thresholds were removed from the dataset. After filtering, samples 1, 2, 3, and 4 had 5,810, 6,065, 5,480, and 5,820 cells, respectively.

Doublet removal was performed using the raw count data using Solo from scvi-tools v1.0.4 (Bernstein et al., 2020; Gayoso et al., 2022). Filtering, including doublet removal, was performed on each individual sample and after integration of the count matrices. For data integration and batch effect removal, we used the single-cell variational inference (scVI) model from scvi-tools. Briefly, the model learns a nonlinear mapping between the latent space and the parameters of a zero-inflated negative binomial distribution used to generate gene expression counts. Batch correction is performed by including batch annotations as an input to the decoder network, allowing the model to learn batch-specific effects that can be removed when sampling from the latent space. Finally, the standard Scanpy processing pipeline including library log-normalization, selection of highly variable genes (*N* = 3,500), dimensional reduction with PCA and UMAP, and clustering was applied to the integrated data. We did not use scVI to regress out covariates due to the likelihood of over-correction in the batch-effect removal step. The resulting cell × gene matrix was 20,081 × 3,500 (24,415 raw). When possible, computational speeds were accelerated using an NVIDIA A30 GPU and the rapids single-cell library v0.10.5, a library for GPU-acceleration of Scanpy processing steps (Avantika et al., 2023).

#### Classification of AAV+ cells

For identification of AAV+ cells, we set a threshold based off the log-normalized expression. Any cell *i* is considered positive for AAV gene *j* if


$$X^{\log ij}>10^{-5}\;\mathrm{where}\;i\in \left\{0,1,2,\ldots ,N\right\}\;\mathrm{and}\;j\in \left\{0,1,2,3\right\}$$

where *N* is the total number of cells minus one and *j* corresponds to each of the four AAV serotypes. By stratifying according to the cell types (Celltype Annotation), we were able to find the cell-specific frequency distribution of all AAV serotypes in the snRNAseq dataset.

#### Cell type annotation

Using the latent representation generated by scVI after integration, a uniform manifold approximation projection (UMAP) was generated. Clusters were generated using the Leiden algorithm at multiple resolutions to capture different size populations and cell type specific markers were used to confirm the identities. Using canonical markers derived from the literature, we identified and annotated each cluster according to the cell type that it belonged to (see Table 1). For clusters that were seemingly distinct but expressed the same cell type clusters, we included a separate metadata column to annotate the clusters with an additional identifier (i.e., mSus and mSus2).

#### Pseudotime and terminal fate probability

We selected only relevant lineages of interest for downstream analysis and confirmation, specifically OSN, Sus, MVC, and BowG lineages. The relevant cell types include HBC, GBC, iOSN, mOSN, iSus, mSus, MVC, and BowG. After subsetting, the nearest neighbor graph was re-calculated again to find the relationships between the remaining clusters. To improve the visualization of our dataset, we used the minimum distortion embedding (MDE) technique available in pyMDE v0.1.184 to generate a new embedding space (Agrawal et al., 2021).

To confirm the identities of our cell type annotations, we determined the pseudotime relationships between clusters using Palantir (Setty et al., 2019) v1.3.3. We specified HBC as the root cell while defining mOSN, mSus, MVC, and BowG as the terminal points for Palantir. We then examined the Palantir-generated terminal fate probabilities of HBC, GBC, and iSus to confirm the annotated cluster identities. The terminal fate probabilities were evaluated with a two-sided *t*-test for each individual lineage and a chi-squared goodness of fit (GOF) to evaluate the total terminal fates with *α* = 0.05.

#### Statistical analyses

Statistical analyses were conducted to assess differences across experimental groups. For Figure 1, data were analyzed using one-way analysis of variance (ANOVA) followed by Tukey’s *post hoc* test to determine significant differences between groups. Results are represented in the figures using compact letter displays, where bars sharing the same letter are not significantly different (*p* > 0.05).

For Figure 2, multiple statistical methods were employed based on the type of data analyzed. For comparisons of terminal fate probabilities of HBCs, a two-sided *t*-test (*α* = 0.05) was used. For evaluating terminal fate probabilities of GBCs and iSus across clusters, a chi-squared goodness-of-fit test was applied. The levels of statistical significance are denoted as follows: ^****^p < 1 × 10^−4^, ^***^p < 1 × 10^−3^, ^**^p < 0.01, ^*^p < 0.05, and ns for not significant. For comparisons of AAV-positive cell percentages across groups, the Kruskal–Wallis test was used to determine significance.

All statistical analyses were performed using GraphPad Prism and Python, and *p*-values less than 0.05 were considered significant unless otherwise stated.

## Data Availability

All code used in bioinformatic analysis is publicly available on Github at https://github.com/LiuzLab/Mouse-AAV-OSN. Raw count matrices and the final processed AnnData object are available with the Github code on Zenodo (https://doi.org/10.5281/zenodo.13620762). Raw FASTQ files are available on the CRN Cloud. All images collected for this manuscript are publicly available through BioImage Archive (Accession S-BIAD1370, DOI: 10.6019/S-BIAD1370).

## Group members of aSCENT-PD Investigators

Benjamin Arenkiel, Zhandong Liu, Brit Mollenhauer, Josef Penninger, Maxime Rousseaux, Armen Saghatelyan, Natalina Salmaso, Christine Stadelmann, Michael G. Schlossmacher, Julianna J. Tomlinson, John M. Woulfe. TCH Neuroconnectivity Core: Ying Wang and Zihong Chen. BCM Single Cell Genomics Core: Mira Jeong.

## References

[ref1] Agrawal A. Ali A. Boyd S. (2021). Minimum-distortion embedding. Found. Trends Mach. Learn. 14, 211–378. doi: 10.1561/2200000090

[ref2] Avantika L. Corey N. J. Rajesh I. Movva R. Miro B. (2023). NVIDIA-genomics-research/rapids-single-cell-examples: v2022.12.0. Available at: https://zenodo.org/records/7566335 (Accessed July 10, 2024).

[ref3] Bernstein N. J. Fong N. L. Lam I. Roy M. A. Hendrickson D. G. Kelley D. R. (2020). Solo: doublet identification in single-cell RNA-Seq via semi-supervised deep learning. Cell Syst. 11, 95–101.e5. doi: 10.1016/j.cels.2020.05.010, PMID: 32592658

[ref4] Brann D. H. Tsukahara T. Weinreb C. Lipovsek M. Van den Berge K. Gong B.. (2020). Non-neuronal expression of SARS-CoV-2 entry genes in the olfactory system suggests mechanisms underlying COVID-19-associated anosmia. Sci. Adv. 6:eabc5801. doi: 10.1126/sciadv.abc5801, PMID: 32937591 PMC10715684

[ref5] Cearley C. N. Wolfe J. H. (2006). Transduction characteristics of adeno-associated virus vectors expressing cap serotypes 7, 8, 9, and Rh10 in the mouse brain. Mol. Ther. 13, 528–537. doi: 10.1016/j.ymthe.2005.11.015, PMID: 16413228

[ref6] Chan K. Y. Jang M. J. Yoo B. B. Greenbaum A. Ravi N. Wu W. L.. (2017). Engineered AAVs for efficient noninvasive gene delivery to the central and peripheral nervous systems. Nat. Neurosci. 20, 1172–1179. doi: 10.1038/nn.4593, PMID: 28671695 PMC5529245

[ref7] Fleming S. J. Chaffin M. D. Arduini A. Akkad A. D. Banks E. Marioni J. C.. (2023). Unsupervised removal of systematic background noise from droplet-based single-cell experiments using cell bender. Nat. Methods 20, 1323–1335. doi: 10.1038/s41592-023-01943-7, PMID: 37550580

[ref8] Fletcher R. B. Das D. Gadye L. Street K. N. Baudhuin A. Wagner A.. (2017). Deconstructing olfactory stem cell trajectories at single-cell resolution. Cell Stem Cell 20, 817–830.e8. doi: 10.1016/j.stem.2017.04.003, PMID: 28506465 PMC5484588

[ref9] Frankish A. Carbonell-Sala S. Diekhans M. Jungreis I. Loveland J. E. Mudge J. M.. (2023). GENCODE: reference annotation for the human and mouse genomes in 2023. Nucleic Acids Res. 51, D942–D949. doi: 10.1093/nar/gkac1071, PMID: 36420896 PMC9825462

[ref10] Gadenstaetter A. J. Schmutzler L. Grimm D. Landegger L. D. (2022). Intranasal application of adeno-associated viruses: a systematic review. Transl. Res. 248, 87–110. doi: 10.1016/j.trsl.2022.05.002, PMID: 35597541

[ref11] Gao G. P. Alvira M. R. Wang L. Calcedo R. Johnston J. Wilson J. M. (2002). Novel adeno-associated viruses from rhesus monkeys as vectors for human gene therapy. Proc. Natl. Acad. Sci. U.S.A. 99, 11854–11859. doi: 10.1073/pnas.182412299, PMID: 12192090 PMC129358

[ref12] Gayoso A. Lopez R. Xing G. Boyeau P. Valiollah Pour Amiri V. Hong J.. (2022). A Python library for probabilistic analysis of single-cell omics data. Nat. Biotechnol. 40, 163–166. doi: 10.1038/s41587-021-01206-w, PMID: 35132262

[ref13] Grimm D. Lee J. S. Wang L. Desai T. Akache B. Storm T. A.. (2008). *In vitro* and *in vivo* gene therapy vector evolution via multispecies interbreeding and retargeting of adeno-associated viruses. J. Virol. 82, 5887–5911. doi: 10.1128/JVI.00254-08, PMID: 18400866 PMC2395137

[ref14] Hammond S. L. Leek A. N. Richman E. H. Tjalkens R. B. (2017). Cellular selectivity of AAV serotypes for gene delivery in neurons and astrocytes by neonatal intracerebroventricular injection. PLoS One 12:e0188830. doi: 10.1371/journal.pone.0188830, PMID: 29244806 PMC5731760

[ref15] Kaminow B. Yunusov D. Dobin A. (2021). STARsolo: accurate, fast and versatile mapping/quantification of single-cell and single-nucleus RNA-seq data. *bioRxiv*. Available at: 10.1101/2021.05.05.442755. [Epub ahead of preprint]

[ref16] Li W. Wu T. Zhu K. Ba G. Liu J. Zhou P.. (2024). A single-cell transcriptomic census of mammalian olfactory epithelium aging. Dev. Cell 59, 3043–3058.e8. doi: 10.1016/j.devcel.2024.07.020, PMID: 39173624

[ref17] Ojala D. S. Sun S. Santiago-Ortiz J. L. Shapiro M. G. Romero P. A. Schaffer D. V. (2018). In vivo selection of a computationally designed SCHEMA AAV library yields a novel variant for infection of adult neural stem cells in the SVZ. Mol. Ther. 26, 304–319. doi: 10.1016/j.ymthe.2017.09.006, PMID: 28988711 PMC5762983

[ref18] Santry L. A. Ingrao J. C. Yu D. L. de Jong J. G. van Lieshout L. P. Wood G. A.. (2017). AAV vector distribution in the mouse respiratory tract following four different methods of administration. BMC Biotechnol. 17:43. doi: 10.1186/s12896-017-0365-2, PMID: 28506256 PMC5433059

[ref19] Setty M. Kiseliovas V. Levine J. Gayoso A. Mazutis L. Pe’er D. (2019). Characterization of cell fate probabilities in single-cell data with Palantir 37, 451–1460. doi: 10.1038/s41587-019-0068-4, PMID: Erratum in: Nat. Biotechnol. 2019. 37(10):1237. doi: 10.1038/s41587-019-0282-0 30899105 PMC7549125

[ref20] Wolf F. A. Angerer P. Theis F. J. (2018). SCANPY: large-scale single-cell gene expression data analysis. Genome Biol. 19:15. doi: 10.1186/s13059-017-1382-0, PMID: 29409532 PMC5802054

